# Selective Detection of Nucleotides in Infant Formula Using an N-Rich Covalent Triazine Porous Polymer

**DOI:** 10.3390/nano12132213

**Published:** 2022-06-28

**Authors:** Yafei Hou, Xiaodan Pei, Yuancheng Wang, Luyuan Zhang, Xiaohui Wei, Hongyan Mao, Wuduo Zhao, Shusheng Zhang, Wenfen Zhang

**Affiliations:** 1 College of Chemistry, Zhengzhou University, Zhengzhou 450001, China; hyf1067653156@163.com (Y.H.); a1483285399@163.com (Y.W.); wxh2421035502@gs.zzu.edu.cn (X.W.); 2 School of Ecology and Environment, Zhengzhou University, Zhengzhou 450001, China; peixiaodan8@163.com (X.P.); zly990502@163.com (L.Z.); 3 Center for Advanced Analysis and Gene Sequencing, Zhengzhou University, Zhengzhou 450001, China; zhaowuduo@163.com (W.Z.); zsszz@126.com (S.Z.); 4 Research Centre of Engineering and Technology for Synergetic Control of Environmental Pollution and Carbon Emissions of Henan Province, Zhengzhou University, Zhengzhou 450001, China

**Keywords:** covalent triazine-based frameworks, solid-phase extraction, nucleotides

## Abstract

The aromatic structure and the rich nitrogen content of polymers based on covalent triazine-based frameworks (CTF) and their unique hydrophilic-lipophilic-balanced adsorption properties make them promising candidates for an adsorbent that can be used for sample pretreatment. Herein, a new covalent triazine-based framework (CTF-DBF) synthesized by a Friedel–Crafts reaction was used for the determination of the content of nucleotides in commercial infant formula. It was shown that the synthetic materials had an amorphous microporous structure, a BET surface area of up to 595.59 m^2^/g, and 0.39 nm and 0.54 nm micropores. The versatile adsorption properties of this material were evaluated by quantum chemistry theory calculations and batch adsorption experiments using five nucleotides as probes. The quantum chemistry results demonstrated that CTF-DBF can participate in multiple interactions with nucleotides. All the analyses performed present good linearity with R^2^ > 0.9993. The detection limits of targets ranged from 0.3 to 0.5 mg/kg, the spiked recoveries were between 85.8 and 105.3% and the relative standard deviations (RSD, n = 6) were between 1.1 and 4.5%. All these results suggest that this versatile CTF-DBF has great potential for sample pretreatment.

## 1. Introduction

Nucleotides identified as conditionally essential nutrients are widely added to infant formula. Studies have shown that the addition of nucleotides to milk powder promotes the growth and maturation of the gastrointestinal tract of infants [1,2]. Consequently, the analysis of nutrients in infant formula should be a useful method to evaluate various infant formulas. There is however no standard method of sample preparation that can achieve effective enrichment of multiple nucleotides in infant formula and at the same time remove matrix interference. With the development of new materials, the development of a new enrichment method may be an effective way to solve this problem.

The unique properties of covalent organic frameworks (COF), including their high surface area, physicochemical stability and structural diversity, have led to their receiving extensive attention during the past few years [3,4,5,6,7,8,9,10]. However, the adsorption by traditional COFs mainly depends on their large conjugated systems, porous structure and hydrophobicity, and this restricts their use with polar or ionic solutes [11,12]. To address this problem, pre-synthetic methods such as modification of the precursor monomers, or post-synthetic steps, including modification of the organic backbone of the COF, were used to introduce specific chemical functionality into the pores of the polymers, tailoring interactions with specific guests while making full use of the surface area and high porosity of the frameworks [13,14,15,16,17,18]. However, both pre-synthetic and post-synthetic modification of COFs generally necessitated tedious synthetic efforts and often harsh experimental conditions and frequently led to side reactions and interfered with the COF formation, all issues that greatly limited the generality of these approaches [13,15,19,20,21,22].

As alternatives to COFs, covalent triazine-based organic framework/-polymers (CTF/CTP) comprised of alternating triazine-benzene units can be synthesized economically under relatively mild conditions [23,24]. The triazine unit and the absence of weak bonds except in the aromatic group endow CTF/CTPs with rich nitrogen content, high chemical and thermal stability, and a remarkable heteroatom effect (HAE) [25,26]. These special properties endow CTFs with useful hydrophilic-lipophilic equilibrium properties, abundant recognition sites and excellent adsorption properties for polar, non-polar and other compounds, which have been applied effectively in the field of adsorption and separation [12,25,27]. For example, Liu et al. [28] prepared a triazine-based covalent organic framework adsorbent with a unique rigid structure by using the trimerization reaction of cyano groups. This rigid structure has multiple adsorption properties, including π-π interaction, hydrophobic interaction and electrostatic attraction. However, all classical CTF syntheses are neither economically nor ecologically favorable, and this seriously limits the applications of CFTs in sample preparation [29,30,31,32,33]. Lars et al. [34] elucidated a solvent-free and time-efficient approach involving Friedel-Crafts alkylation for the synthesis of porous CTFs. Its application as a sorbent is still limited, however, and its mechanism of adsorption is also unclear. Thus, it is necessary to expand the value of sample pretreatment and to investigate the adsorption mechanism.

Herein, we describe CTF-DBF, a new type of CTF synthesized according to the method proposed by Lars. This uses dibenzofuran (DBF) and cyanuric chloride (2,4,6-trichloro-1,3,5-triazine, CC) as monomers. The morphology and physicochemical properties of CTF-DBF were characterized by SEM, elemental analysis, FT-IR spectra, solid-state ^13^C NMR, nitrogen sorption analysis and thermogravimetric analysis. Nucleotides are compounds with a nitrogenous base and an aldopentose and at least one phosphate group, and triazines are aromatic heterocyclic rings containing N atoms and a π-electron structure. Accordingly, the proposed CTF-DBF has several possible interactions with nucleotides, including hydrogen bonding, hydrophobic interactions, electrostatic attraction, and π-π interaction and, as a result, could be an efficient solid phase-extraction (SPE) adsorbent for nucleotides. Five nucleotides were selected as probes to evaluate the adsorption capacity and mechanism of CTF-DBF. Quantum chemistry theory calculations were conducted to describe quantitatively the versatile adsorption properties of CTF-DBF. In addition, adsorption experiments were conducted to identify five nucleotides in the infant formula. The main factors that influence the SPE efficiency, such as the amount of adsorbent, composition, and volume of desorption solvent, times reused, and sample pH, were optimized, leading to a CTF-DBF with the potential to become a powerful food sample pretreatment adsorbent.

## 2. Experimental Section

### 2.1. Materials and Instruments

**Figure 1:**
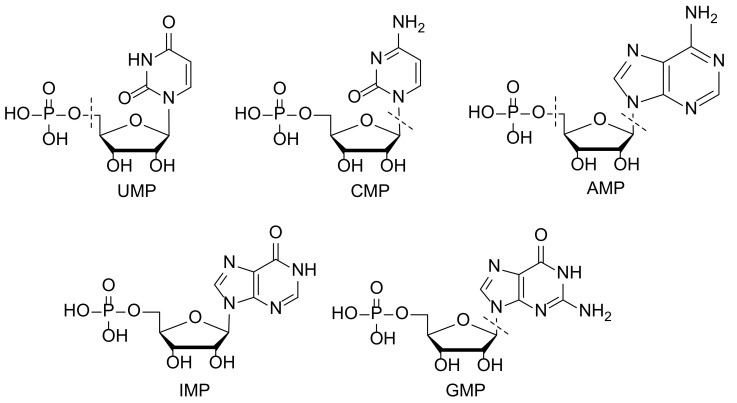
Chemical structural formulas of five nucleotides.

Aluminum trichloride, cyanuric chloride and dibenzofuran were purchased from Aladdin Reagent Co., Ltd. (Shanghai, China). Analytical grade 1,2-dichloroethane, ethanol, tetrahydrofuran, methanol, acetone, and other solvents were provided by Sinopharm Chemical Reagent Co., Ltd. (Shanghai, China). Uridine 5′-monophosphate (UMP), cytidine 5′-monophosphate (CMP), adenosine 5′-monophosphate (AMP), inosine 5′-monophosphate (IMP), and guanosine 5′-monophosphate (GMP) standards (Figure 1) (purity ≥ 98%) were purchased from Aladdin Biochemical Technology Co., Ltd. (Shanghai. China). A 3 mL polypropylene solid-phase extraction empty column tube and a matching 20 μm pore size PTFE sieve plate were purchased from Waters (Framingham, Massachusetts, USA). The ultrapure water used in the experiment was purified from a Milli-Q system. Infant formula samples were purchased from different local supermarkets in Zhengzhou.(Zhengzhou, Henan, China). 

Scanning Electron Microscope (SEM) images were collected on an S-4300 SEM configured with an energy dispersive spectrometer system (Zeiss/Auriga FIB, Oberkochen, Baden-Württemberg, Germany). Solid-state ^13^C NMR spectra were performed at ambient pressure on a Bruker AVANCE 400 spectrometer (Bruker, Bly red card, MA, USA) using a standard Bruker magic angle-spinning probe with 4 mm (outside diameter) zirconia rotors. Transmission electron microscopy (TEM) images were produced on a TalosF200S TEM (Thermo Fisher, Waltham, MA, USA). A Nicolet 6700 spectrometer (Shimadzu, Kyoto, Japan) was used to record the Fourier-transform infrared (FT-IR) spectra. N_2_ adsorption/desorption isotherms were measured on an Autosorb-1 MP gas adsorption instrument (Quantachrome, Boynton, FL, USA). Elemental analysis was accomplished with a Flash EA 1112 elemental analyzer (Thermo Electron, Waltham, MA, USA). X-ray diffraction (XRD) spectra were collected from an ARL X′TRA diffraction-meter using Cu Kα radiation (ARL, Lausanne, Switzerland). Ultrapure water use in this work was taken from a Milli-Q gradient ultrapure water system (Millipore, Boston, MA, USA). The HPLC separations were performed on Agilent 1260 (Agilent, Palo Alto, CA, USA) with an Ultimate XB-C18 column (250 × 4.6 mm i.d., 5 μm) and a UV detector.

### 2.2. Synthesis of Porous Covalent Triazine Framework Materials

**Figure 2:**
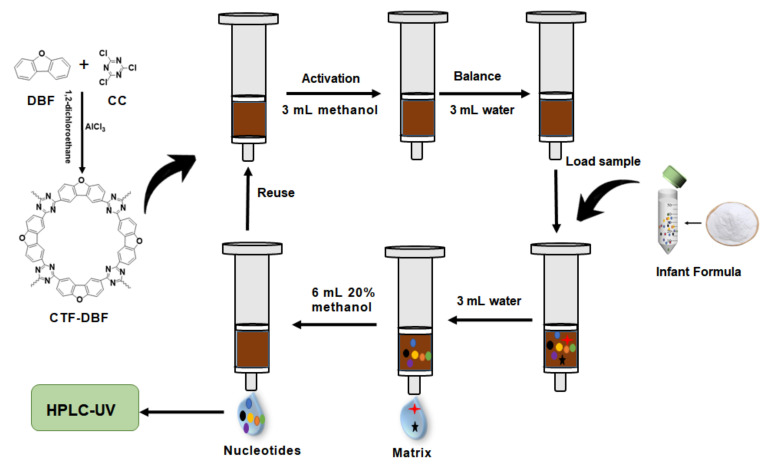
Schematic of this work.

The synthesis was conducted using published methods with some modifications. Briefly, dibenzofuran (2.52 g) dissolved in 1,2-dichloroethane (100 mL) was added dropwise to a mixture of cyanuric chloride (1.84 g) and aluminum trichloride (4.80 g) and the mixture was refluxed under a nitrogen atmosphere for 24 h. After cooling to room temperature (rt), the product was washed thoroughly with ice water, tetrahydrofuran, and ethanol three times to obtain a crude product. Soxhlet extraction was conducted for 48 h with a mixture of tetrahydrofuran and methanol as the extraction liquid to further purify the obtained product. The final product was vacuum dried for 12 h at 60 °C to obtain CTF-DBF as a brown powder. The yield of polymer is up to 85%. A schematic of this method is shown in Figure 2.

### 2.3. Quantum Chemistry Calculations

All calculations were performed using the Gaussian 09 program. To explore the adsorption mechanism of the CTF-DBF adsorbent and nucleotides, their optimized molecular structures and adsorption energies of the nucleotides were obtained using density functional theory (DFT). Molecular docking was conducted using the AutoDock Vina program to help obtain the rational preliminary estimates of complexes of various nucleotides with CTF-DBF. The lowest energy structure of each complex was selected for further optimization at the B3LYP/6-31G (d,p) level. Finally, molecular non-covalent interaction (NCI) analysis was conducted by using the Multiwfn program. The stabilization energy (△E_n_), calculated from Equation (1) changes on the formation of a so-called CTF-DBF-analyte supramolecule:

△E_n_ = E_CTF-DBF-n_ − E_CTF-DBF_ − E_n,_ (1)

where E_CTF-DBF-n_ is adsorption energy between CTF-DBF and the target molecule, E_CTF-DBF_ is energy of CTF-DBF and E_n_ is the energy of the target. 

### 2.4. Sample Pretreatment

Infant formula milk powder (5 g) was dissolved into ultrapure water (20 mL) at 50–60 °C and the solution was cooled to rt. The obtained solution was first ultrasonicated for 10 min, then the pH was adjusted to 4.0 with acetic acid. The solution was then diluted to 50 mL and filtered for use in the following experiments.

SPE measurements were performed to verify the adsorption capacity of the prepared CTF-DBF adsorbent. Briefly, the above filtrate (5 mL) was loaded onto a self-made 80 mg/3 mL CTF-DBF solid-phase extraction cartridge which had been pretreated with methanol (3 mL) and water (3 mL). Then the nucleotides were eluted with a 20% methanol aqueous solution (6 mL). The eluent was evaporated to dryness at rt under a gentle stream of N_2_ and the residues were redissolved in the mobile phase (1 mL).

### 2.5. Detection Conditions

An Agilent 1260 with a UV detector was used to perform all the analyses in the experiments. A unitary C18 column (250 × 4.6 mm I.D., 5 μm) was used as the chromatographic separation column. The injection volume was set at 20 μL, the column temperature at 30 °C, the detection wavelength at 254 nm, and the flow rate at 0.8 mL/min. The mobile phase was composed of an aqueous solution of MeOH/KH_2_PO_4_ (10/90, *v*/*v*).

## 3. Results and Discussion

### 3.1. Material Characterization of CTF-DBF

**Figure 3:**
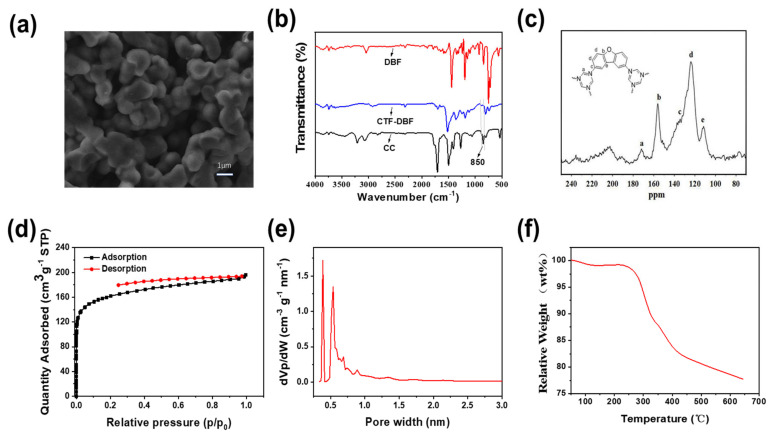
Characterization of CTF-DBF: (**a**) SEM; (**b**)FT-IR; (**c**) Solid-state ^13^C NMR; (**d**) N_2_ adsorption-desorption isotherms; (**e**) Pore size distribution; (**f**) TGA.

The external morphology of the prepared CTF-DBF material was first characterized and analyzed with a scanning electron microscope. As shown in Figure 3a, the CTF-DBF polymer consists of a series of aggregated particles, the smaller ellipsoidal ones ranging from 1 to 1.5 μm in size. The chemical structure of the networks was examined by Fourier transform infrared (FT-IR) spectroscopy. As shown in Figure 3b, compared with cyanuric chloride, the intensity of the C-Cl stretching vibration absorption peak at 850 cm^−1^ in CTF-DBF is significantly reduced, indicating the completion of the Friedel–Crafts alkylation reaction shown in Figure 2. The presence of characteristic C-O-C vibrations (1191 cm^−1^) of the furan unit and the strong C=N vibrations (1521 cm^−1^and 1365 cm^−1^) confirm the existence of the triazine core (Figure 3b). All those FT-IR results support the successful formation of the network. The network formation was further confirmed by solid-state ^13^C NMR. As shown in Figure 3c, the resonance absorption peak at 172 ppm corresponds to the triazine ring carbons, strongly supporting the presence of the cyanuric chloride moiety in the network. All the other major peaks, ranging from 110 to 160 ppm, may be attributed to the aromatic carbons of the dibenzofuran. All the signals show little shift, which is due to the electron-withdrawing effect of triazine moieties and thus a changing electronic environment of the carbon atoms in CTF-DBF. The above data further prove that the porous CTF-DBF material had been successfully prepared.

The porosity and the high surface area of CTF-DBF were investigated by N_2_ adsorption analysis. The N_2_ isotherms of CTF-DBF were measured at 77 K. As shown in Figure 3d, the microporosity of CTF-DBF is implied by the steep increase of the N_2_ isotherms in the low relative pressure range. A continued gentle rise in N_2_ in the entire pressure range may be attributed to the mesopores with a very broad size distribution or the outer surfaces of small particles. Thus, the isotherm curve follows a combination of types I and II in the IUPAC classification. The hysteresis loop indicates the flexible framework structure of CTF-DBF.A Brunauer–Emmet–Teller (BET) model and the quenched solid density functional theory (QSDFT) method were conducted to investigate the specific surface area and the pore size distribution, respectively. The physisorption experiments reveal an intrinsic and permanent surface area of 595.59 m^2^/g and a sharp pore size distribution (Figure 3e) with two micropores of 0.39 nm and 0.54 nm. 

Thermogravimetric analysis (TGA) was used to explore the thermal stability of the material, and the result is shown in Figure 3f. It can be seen that the mass of CTF-DBF decreased by 2% below 250 °C. This may be caused by the material absorbing some moisture and indicates that the material is relatively stable below 250 °C and the structure does not change. As the temperature increases, the material begins to decompose above 250 °C, so the temperature at which CTF-DBF is used should be below 250 °C to ensure that the material is stable and has better adsorption.

**Figure 4:**
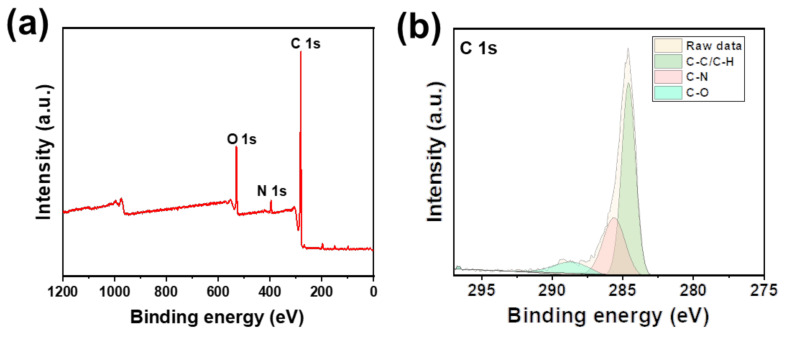
XPS spectra of CTF-DBF: (**a**) Wide-scan survey spectra; (**b**) High-resolution C 1s.

To gain insight into the local chemical environment, X-ray photoelectron spectroscopy (XPS) was conducted. The low-resolution XPS spectra demonstrated the existence of C 1s, O 1s, and N 1s electrons (Figure 4a). The high-resolution XPS spectra of C 1s reveal three major peaks (Figure 4b), which can be attributed to sp^2^ carbon atoms of the dibenzofuran moiety (284.6 eV), the carbon atoms in the triazine node (285.8 eV), and the carbon atoms of C-O-C (288.7 eV) of the oxygens in CTF-DBF. All the above results further confirmed that the polymer had been successfully prepared.

### 3.2. Study of Intermolecular Forces

To describe the interactions between CTF-DBF and CMP, UMP, IMP, AMP, and GMP directly and quantifiably, the optimized complex with the supramolecular structures of analyte-CTF-DBF at the lowest energy was first obtained using quantum chemistry calculations in vacuo and DFT/B3LYP/6-31G (d,p) as the basis set (Figure S1). As the stabilization energy (△E_n_) changes (Table S1) upon the formation of a CTF-DBF-analyte complex, we also calculated △E_n_ using Equation (1). From the results of quantum chemistry calculation, it can be seen that all the adsorption energies between CTF-DBF and guest molecules are negative, which implies that these so-called supramolecules are stable. In addition, the adsorption energies of CMP and GMP are significantly higher than those of IMP and UMP, which is due to the existence of amino groups which significantly enhance the hydrogen bonding interaction between CTF-DBF and guest molecules. However, the △E of IMP is higher than that of the nitrogen-rich compound UMP and why the significantly different structures compounds (UMP, CMP, AMP) should have similar △E values. This may be the result of the anisotropic of the multiple forces such as inclusion interactions, π-π interactions, and hydrogen bonding interactions generated among CTF-DBF and detection targets. For example, the hydrogen bonding of UMP, CMP, and AMP increased with the increase of nitrogen content and amino group number, while their inclusion decreased with the increase of molecular size, which led to similar △E values.

**Figure 5:**
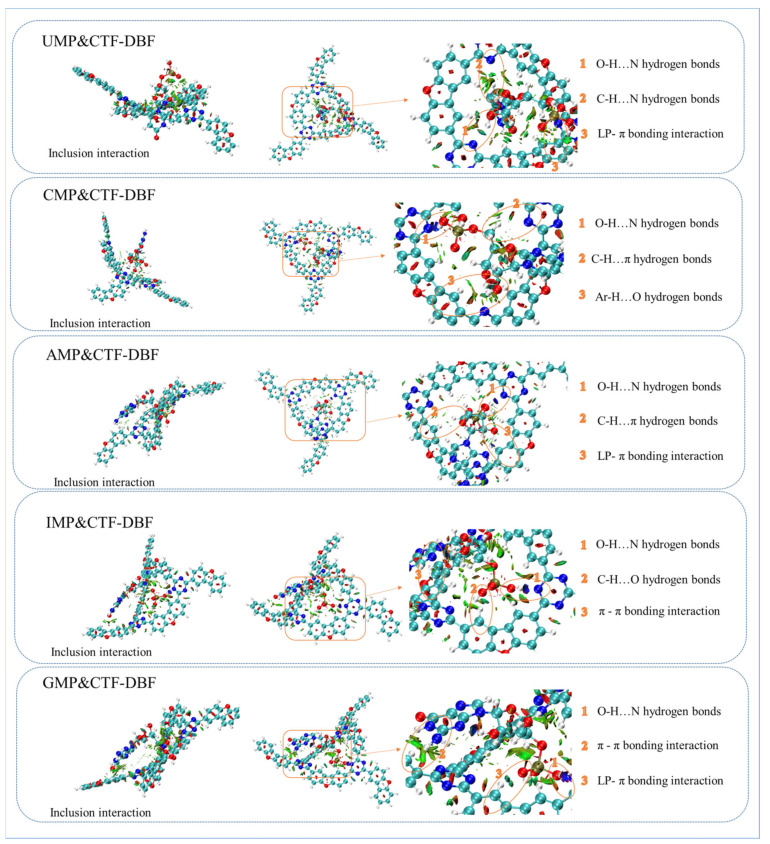
Theoretical analysis of non-covalent forces.

To further confirm this inference, a non-covalent interaction (NCI) was conducted as a theoretical research strategy to explore the detailed intermolecular interactions between CTF-DBF and nucleotides. As shown in Figure 5, nucleotides with various structures can generate π-π interactions, CH…π interactions and NH...O hydrogen bonds with CTF-DBF. For example, IMP can insert into the cavity of CTF-DBF using an inclusion interaction; the H_2_PO_4_^−^ in nucleotides can insert into or approach the cavity of CTF-DBF, forming O-H…N hydrogen bonds with the nitrogen heterocycle of CTF-DBF. The benzene ring and partially protonated nitrogen can control the formation and assembly of the supramolecular structure of the complex by forming hydrogen bond interactions and π-π interactions, while the benzene ring, nitrogen heterocycle, and electron-donating amino groups promote the formation of CH...π interactions, CH…O, OH…N, NH...O hydrogen bonds, and LP...π interactions. NCI analysis intuitively clarified the excellent adsorption potential of CTF-DBF for nucleotides from the theoretical perspective.

### 3.3. Optimization of Solid-Phase Extraction Conditions

**Figure 6:**
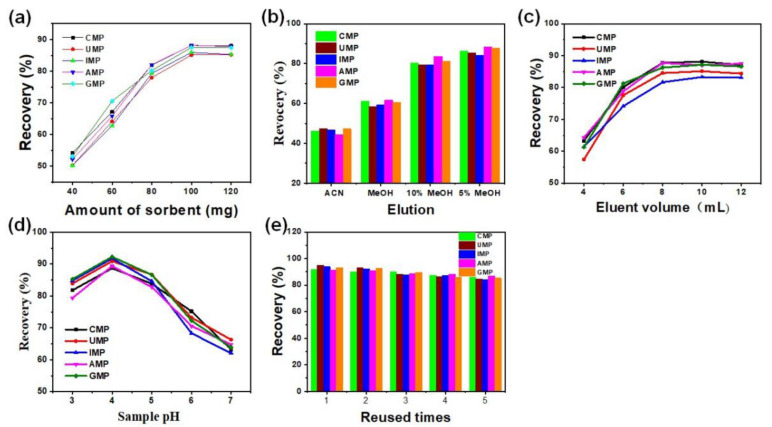
Optimization of solid-phase extraction conditions: (**a**) Amount of sorbent; (**b**) Type and composition of eluate; (**c**) Elution volume; (**d**) Sample pH; (**e**) Reused times.

An effective and sensitive SPE-HPLC method was developed for nucleotides in infant formula using CTF-DBF as a solid-phase extraction (SPE) adsorbent for enrichment and purification. The main factors, including the amount of adsorbent, sample pH, composition and volume of desorption solvent and the extent of reuse, all affect the extraction efficiency. All these main factors were investigated and evaluated in the sample pretreatment step to obtain the best extraction efficiency using 5 mL of a 100 μg/mL nucleotides standard solution sample as the test probe. The amount of sorbent is closely correlated with the quantity of adsorbed analytes, so the experiments were first studied with the sorbent amount ranging from 40 mg to 120 mg. As can be seen in Figure 6a, 100 mg was sufficient to achieve a satisfactory extraction efficiency and, accordingly, 100 mg was selected as the optimal adsorbent amount. When the dosage of the adsorbent ranged from 40 mg to 120 mg, the recovery rate of GMP maintained a high rate, corresponding to the results in Table S1. The proper type and composition of eluate play an important role in improving the recovery of substances and reducing the interface. Thus, the nature and composition of the eluate were optimized (Figure 6b) and maximum extraction efficiency was obtained with a mixture of water: MeOH (5:95, *v*/*v*). For the choice of the elution volume, varying volumes (4 to 12 mL) of 5% MeOH were applied for solid-phase extraction progress. As shown in Figure 6c, the recoveries increase with the increase of elution volume from 4 to 8 mL and remain almost constant when the elution volume increases from 8 mL to 12 mL. Therefore, 8 mL of 5% MeOH solution was chosen for the following experiments: when the sample pH varied from pH 3 to 7 (Figure 6d), the recovery increased from pH 2 to 4 and significantly decreased from pH 4–7 and the optimal sample loading pH was 4. Moreover, as can be seen from Figure 6e, the collected composites can be reused five times without any obvious loss of performance.

### 3.4. Methodology Verification

Method validation methods, including limit of quantification (LOQ), limit of detection (LOD), linear range, and correlation coefficient (r^2^) were studied and are summarized in Table S2. The results showed that good linearity was obtained in the range of 1–100 μg/mL for UMP, 1.5–50 μg/mL for IMP, 2–100 μg/mL for CMP and AMP with the correlation coefficients all being >0.9993. LODs (S/N = 3) for the analytes were between 0.30 and 0.50 pg/mL and LOQs (S/N = 10) were between 1.10 and 1.70 pg/mL.

A recovery study was performed to validate the accuracy of the developed method. Spiked samples at 2, 10 or 50 mg/kg were prepared by adding a known amount of a solution of standard nucleotides to the blank infant formula milk powder matrix. As shown in Table 1, the recoveries ranged from 85.8 to 105.3% with RSD values ranging from 1.1 to 4.5%, which demonstrated that the developed SPE-HPLC method possesses good accuracy. The chromatograms of blank samples and spiked samples treated by solid-phase extraction are shown in Figure S2.

### 3.5. Actual Sample Determination

The applicability of the proposed method was investigated by analyzing actual samples. Eight infant formula milk powders with added nucleotides described in the nutrition ingredient tables of different brands were determined using this developed method. As shown in Table 2, the amount of each of the five nucleotides determined using the developed method was equivalent to the added amount indicated in the nutrient composition table printed on the outer packaging. This further proved that the method has a certain accuracy and can meet the detection requirements of actual samples.

## 4. Conclusions

In summary, a new type of covalent triazine-based framework (CTF) has been synthesized by a one-pot method. Its morphology and physicochemical properties were measured by SEM, FT-IR spectra, solid-state ^13^C NMR, nitrogen sorption analysis, and thermogravimetric analysis. The results demonstrated its high physicochemical stability and excellent absorption affinity for nucleotides. Quantum chemistry calculation results further demonstrated that CTF-DBF, a versatile covalent triazine framework material, can have various interactions with nucleotides, including inclusion interaction, π-π interactions, CH…O, OH…N, NH...O hydrogen bond interactions, CH...π interactions and LP...π interactions, which ensure the adsorption capacity to nucleotides. Under the optimized SPE conditions, a new method for simultaneous determination of five nucleotides in infant formula by SPE-HPLC was established. The detection limit of the target ranges from 0.3 to 0.5 mg/kg and the recovery ranges from 85.8 to 105.3% with an RSD between 1.1 and 4.5%. However, the selectivity of the adsorbent constructed in this paper is relatively poor, so we seek to modify it in the follow-up research aimed at improving its molecular recognition performance and achieving the enrichment detection of specific targets.

## Figures and Tables

**Table 1:** Recovery and precision of 5 different nucleotides (n = 6).

Analytes	Spiked Level (mg/kg)	Detection Value (mg/kg)	Recovery (%)	RSD (%)
CMP	2.0	1.76	88.2	2.1
	10.0	9.17	91.7	1.7
	50.0	45.15	90.3	3.4
UMP	1.0	0.87	87.6	4.3
	5.0	5.11	102.1	2.2
	25.0	26.15	104.6	3.1
IMP	1.0	0.86	85.8	2.6
	5.0	4.36	87.2	1.1
	25.0	22.10	88.4	3.5
AMP	2.0	1.76	88.1	4.5
	10.0	10.53	105.3	3.4
	50.0	51.35	102.7	2.7
GMP	2.0	1.73	86.6	2.4
	10.0	8.71	87.1	2.2
	50.0	44.71	89.4	1.6

**Table 2:** Contents of five nucleotides in real samples.

Sample No	CMP (mg/kg)	UMP (mg/kg)	IMP (mg/kg)	AMP (mg/kg)	GMP (mg/kg)	Total Content (mg/kg)	Labeled Content (mg/kg)
1	69.7	39.5	26.1	47.6	13.4	196.3	197
2	84.2	70.6	17.8	51.6	17.4	241.6	240
3	61.5	36.2	16.7	24.1	13.5	152.0	150
4	79.1	63.8	25.4	44.7	15.8	228.8	230
5	123.2	82.4	21.3	53.5	17.6	298.0	300
6	57.8	41.7	15.1	25.5	13.5	153.6	153
7	117.5	77.6	18.6	64.5	20.3	298.5	300
8	98.1	65.7	19.8	48.2	17.9	249.7	250

## Supplementary Materials

The following supporting information can be downloaded at: https://www.mdpi.com/article/10.3390/nano12132213/s1, Figure S1: (a) Optimal structure of the host molecule CTF-DBF; (b-f)Optimal conformation of host and guest molecules; the optimization of the above structural models all use the B3LYP/6–31+G basis set; carbon atoms are gray, hydrogen atoms are white, oxygen atoms are red, nitrogen atoms are blue, and chlorine atoms are green; Figure S2: Chromatograms of five nucleotides after SPE treatment of blank and blank spiked samples (a:Blank sample; b:Blank spiked sample 5.0 mg/kg); Table S1: Adsorption energy between host and guest molecules ΔEn; Table S2: Relevant parameters of SPE-HPLC-UV metho;The coordinates of the CTF-DBF.
