# Supplementary material for: Selective Detection of Nucleotides in Infant Formula Using an N-Rich Covalent Triazine Porous Polymer
Source: Nanomaterials (Basel). 2022 Jun 28;12(13):2213. doi: 10.3390/nano12132213 (PMC9268561; doi:10.3390/nano12132213)
Supplement: Supplementary file 1 [file nanomaterials-12-02213-s001.zip › nanomaterials-1689532-supplementary.pdf]

Supplementary Material

Selective Detection of Nucleotides in Infant Formula Using an N-Rich Covalent Triazine Porous Polymer

Yafei Hou [1,†], Xiaodan Pei [2,†], Yuancheng Wang [1], Luyuan Zhang [2], Xiaohui Wei [1], Hongyan Mao [1,*], Wuduo Zhao [3], Shusheng Zhang [3] and Wenfen Zhang [1,4,*]

1 College of Chemistry, Zhengzhou University, Zhengzhou 450001, China; hyf1067653156@163.com (Y.H.); a1483285399@163.com (Y.W.); wxh2421035502@gs.zzu.edu.cn (X.W.)
2 School of Ecology and Environment, Zhengzhou University, Zhengzhou 450001, China; peixiaodan8@163.com (X.P.); zly990502@163.com (L.Z.)
3 Center for Advanced Analysis and Gene Sequencing, Zhengzhou University, Zhengzhou 450001, China; zhaowuduo@163.com (W.Z.); zsszz@126.com (S.Z.)
4 Research Centre of Engineering and Technology for Synergetic Control of Environmental Pollution and Carbon Emissions of Henan Province, Zhengzhou University, Zhengzhou 450001, China
* Correspondence: mhy70@zzu.edu.cn (H.M.); zhangwenfen1988@126.com (W.Z.); Tel.: +0371-67739689 (W.Z.)
† These authors contributed equally and should be considered co-first authors.

Table S1. Adsorption energy between host and guest molecules ΔEn.

CTF-DBF-analytes	△En / (kJ/mol)
CTF-DBF-UMP	−42.01
CTF-DBF-CMP	−55.60
CTF-DBF-AMP	−45.55
CTF-DBF-IMP	−27.95
CTF-DBF-GMP	−133.99

Note: Using the B3LYP method combined with the 6-31G basis.

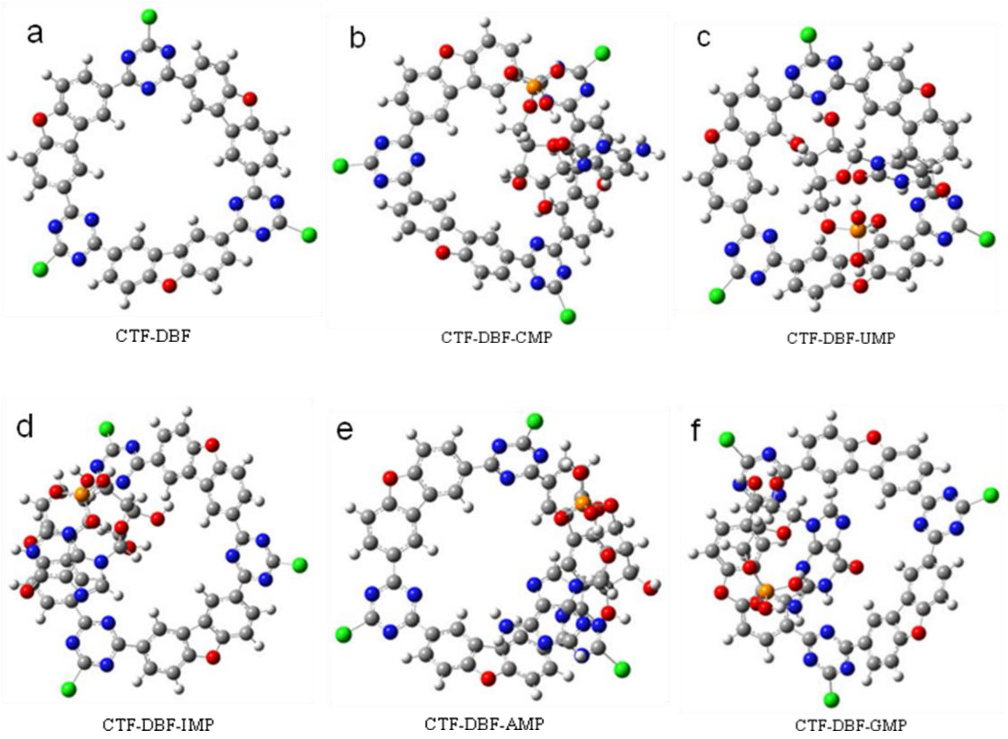

Figure S1. (**a**) Optimal structure of the host molecule CTF-DBF;(**b–f**)Optimal conformation of host and guest molecules;the optimization of the above structural models all use the B3LYP/6–31+G basis set; carbon atoms are gray, hydrogen atoms are white, oxygen atoms are red, nitrogen atoms are blue, and chlorine atoms are green.

Table S2. Relevant parameters of SPE-HPLC-UV method.

**Analytes**	**Linear range/ (μg/mL)**	**Regression equation**	***r***	**LOD/ (mg/kg)**	**LOQ/ （mg/kg）**
CMP	2–100	y = 27.921x + 1.224	0.9998	0.50	1.70
UMP	1–100	y = 15.103x + 0.335	0.9998	0.30	1.20
IMP	1.5–50	y = 13.036x + 0.049	0.9996	0.50	1.50
AMP	2–100	y = 68.225x + 1.446	0.9995	0.30	1.20
GMP	1–100	y = 71.016x − 1.645	0.9993	0.30	1.10

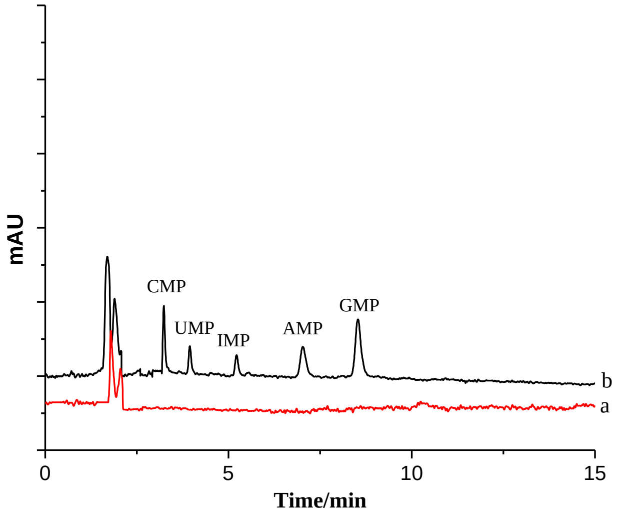

Figure S2. Chromatograms of five nucleotides after SPE treatment of blank and blank spiked samples. (a: Blank sample; b: Blank spiked sample 5.0 mg/kg).

The coordinates of the CTF-DBF:

```
C 0.34192567 -4.86872164 -1.38669058
C 0.63673900 -5.80865674 -2.39955546
C 1.70970866 -5.63705625 -3.26274054
C 2.49193365 -4.50925822 -3.06282748
C 2.21910997 -3.52865083 -2.08669807
C 1.11636975 -3.70758110 -1.24530331
H 0.01130267 -6.68878683 -2.47744519
H 1.95105685 -6.35790970 -4.03552016
H 0.87348826 -2.99886465 -0.46131185
C 4.15456207 -3.08910461 -3.20205943
C 5.37214144 -2.52924411 -3.55629904
C 5.76238072 -1.39905651 -2.84979251
C 4.93353353 -0.82003693 -1.86475903
C 3.69682956 -1.39279545 -1.54294514
C 3.31580096 -2.57184370 -2.19398566
H 5.98980459 -2.96954362 -4.33062582
H 6.71914276 -0.93400146 -3.05564056
H 3.07249744 -0.93647954 -0.78206512
O 3.64517255 -4.23908140 -3.75248989
C -0.73921053 -5.18896089 -0.42471465
```

N -1.24883608 -4.20943170 0.34274503
C -2.17314454 -4.60792254 1.23391263
C -2.02452529 -6.78036439 0.57260758
N -2.56726367 -5.88022627 1.40337609
N -1.11735784 -6.47559391 -0.36565805
C -2.84975402 -3.59648022 2.07992618
C -3.55335369 -4.04182469 3.22142066
C -2.87031146 -2.24244806 1.71368532
C -4.30651100 -3.17261881 3.99794378
H -3.50847039 -5.09420597 3.47115018
C -3.64658982 -1.35470880 2.46558584
H -2.32394949 -1.92132370 0.83373696
C -4.35415598 -1.85096337 3.58002385
H -4.85851631 -3.50639605 4.86910572
C -4.03441810 0.04858032 2.36474021
C -4.95868021 0.24813857 3.41032091
C -3.74834337 1.11805301 1.50819386
C -5.64901174 1.43256773 3.61533214
C -4.45064086 2.31772732 1.67739337
H -3.02312612 1.02587034 0.70661577
C -5.40009962 2.45805131 2.71245556
H -6.35924037 1.53630892 4.42761277
H -5.93322305 3.39750036 2.79783393
O -5.12961998 -0.88479992 4.16652067
C -1.55064507 5.53584687 -0.50866250
C -1.45992350 6.92639800 -0.74241132
C -0.39041847 4.75204792 -0.41408030
C -0.23469896 7.57177394 -0.83209895
H -2.37981990 7.49210504 -0.81748407
C 0.85466516 5.37633972 -0.54167303
H -0.47940190 3.68813986 -0.22467422
C 0.89379882 6.77631571 -0.71036147
H -0.15435471 8.64329167 -0.97484524
C 2.25473642 4.96129695 -0.50509895
C 2.99271690 6.15982416 -0.59907044
C 2.95078843 3.74951392 -0.44611856
C 4.37696061 6.21979333 -0.56262374
C 4.35369225 3.77780245 -0.43115741
H 2.43610455 2.79525040 -0.43190675
C 5.04687754 5.00857627 -0.46243711
H 4.90023948 7.16706336 -0.62476807
H 6.12899768 4.98514860 -0.44105618

O 2.17850420 7.25456589 -0.73447811

C 5.43725586 0.40380171 -1.19181568

N 4.58088749 1.40424761 -0.92696829

C 5.16208207 2.53588693 -0.48526452

C 7.21754312 1.56233051 -0.38364319

N 6.46111001 2.64741308 -0.16795373

N 6.75690168 0.43801855 -0.95241666

C -2.89924209 4.96796853 -0.26932577

N -3.00167540 3.79626447 0.38606028

C -4.24679265 3.48614534 0.78415400

C -5.15518268 5.27458216 -0.28415911

N -5.34689905 4.19408623 0.48837911

N -3.94548209 5.71593615 -0.65359123

C 8.64202630 1.61040449 0.01352842

C 9.47267498 0.49179159 -0.22131950

C 9.16000071 2.76032194 0.62687370

C 10.81625777 0.49343147 0.13851162

H 9.03460835 -0.38026512 -0.69156156

C 10.50375156 2.77939144 0.99625131

H 8.50706991 3.60659486 0.80471495

C 11.30293383 1.64619079 0.74232469

H 11.45841647 -0.36179650 -0.03887086

C 11.38962285 3.74003641 1.63204966

O 12.59423919 1.83122507 1.16498251

C 12.64141428 3.10157798 1.70349759

C 11.26099081 5.03865155 2.13627575

C 13.77083009 3.69173784 2.25109651

C 12.38292866 5.65176637 2.69154570

H 10.30829195 5.55779783 2.09703982

C 13.61930934 4.98784024 2.74776734

H 14.71842620 3.16621206 2.28779355

H 14.47578473 5.49062608 3.18619545

C -6.34719804 6.02426863 -0.73831283

C -6.19270782 7.16593849 -1.55589440

C -7.62697501 5.59283238 -0.35953235

C -7.28895807 7.89450221 -2.00559338

H -5.18944999 7.46544731 -1.83351119

C -8.73722005 6.31103174 -0.79983443

H -7.72530398 4.71138890 0.26311182

C -8.54403019 7.44672481 -1.61230123

H -7.17669614 8.77054113 -2.63425049

C -10.17650343 6.19665622 -0.63490875

O -9.73485294 8.03352306 -1.95549333
C -10.72107338 7.27202784 -1.36060015
C -11.04065337 5.32151082 0.03183816
C -12.08251579 7.51957463 -1.45498722
C -12.41320120 5.55071929 -0.04908343
H -10.65134497 4.48176651 0.59921648
C -12.92602464 6.63380653 -0.78159217
H -12.46340832 8.35970103 -2.02470108
H -13.99982323 6.78708224 -0.82674449
C -2.44986237 -8.19309138 0.69792504
C -3.48561760 -8.53836609 1.59441439
C -1.82339222 -9.18303580 -0.07285324
C -3.91566389 -9.85307458 1.74133616
H -3.94909151 -7.74664194 2.17017274
C -2.23901346 -10.50656271 0.06255173
H -1.02792550 -8.90121515 -0.75235464
C -3.27651072 -10.81263909 0.96628216
H -4.71238714 -10.12286068 2.42523552
C -1.85735696 -11.77770398 -0.52939840
O -3.56130067 -12.15413194 0.97648335
C -2.69851174 -12.73428341 0.06829131
C -0.91818973 -12.19838354 -1.47712506
C -2.65129335 -14.08778080 -0.23101203
C -0.85258264 -13.55428758 -1.79365754
H -0.25427000 -11.48495254 -1.95556829
C -1.70674241 -14.48480228 -1.17953717
H -3.31725700 -14.79415141 0.25169096
H -1.63263649 -15.53456214 -1.44615190
H -0.12981830 -13.89889109 -2.52660901
H 12.30173339 6.65901241 3.08804314
H -13.09893368 4.88185845 0.46154237
